# Current Therapeutic Approaches for Multidrug-Resistant and Extensively Drug-Resistant *Acinetobacter baumannii* Infections

**DOI:** 10.3390/antibiotics13030261

**Published:** 2024-03-15

**Authors:** Petros Rafailidis, Periklis Panagopoulos, Christos Koutserimpas, George Samonis

**Affiliations:** 1 Second University Department of Internal Medicine, University General Hospital of Alexandroupolis, 681 00 Alexandroupolis, Greece; prafaili@med.duth.gr (P.R.); ppanago@med.duth.gr (P.P.); 2 Department of Orthopaedics and Traumatology, “251” Hellenic Air Force General Hospital of Athens, 115 25 Athens, Greece; chrisku91@hotmail.com; 3 Department of Oncology, Metropolitan Hospital, 185 47 Athens, Greece; 4 Department of Medicine, University of Crete, 715 00 Heraklion, Greece

**Keywords:** *A. baumannii*, antimicrobial resistance, sulbactam, colistin, polymyxin B, tigecycline, cefiderocol

## Abstract

The treatment of *Acinetobacter baumannii* infections remains a challenge for physicians worldwide in the 21st century. The bacterium possesses a multitude of mechanisms to escape the human immune system. The consequences of *A. baumannii* infections on morbidity and mortality, as well on financial resources, remain dire. Furthermore, *A. baumannii* superinfections have also occurred during the COVID-19 pandemic. While prevention is important, the antibiotic armamentarium remains the most essential factor for the treatment of these infections. The main problem is the notorious resistance profile (including resistance to carbapenems and colistin) that this bacterium exhibits. While newer beta lactam/beta-lactamase inhibitors have entered clinical practice, with excellent results against various infections due to Enterobacteriaceae, their contribution against *A. baumannii* infections is almost absent. Hence, we have to resort to at least one of the following, sulbactam, polymyxins E or B, tigecycline or aminoglycosides, against multidrug-resistant (MDR) and extensively drug-resistant (XDR) *A. baumannii* infections. Furthermore, the notable addition of cefiderocol in the fight against *A. baumannii* infections represents a useful addition. We present herein the existing information from the last decade regarding therapeutic advances against MDR/XDR *A. baumannii* infections.

## 1. Introduction

*Acinetobacter baumannii* (*A. baumannii*) received its name in 1954 (almost 40 years after its initial description by Beijerink). It gradually became evident that this immotile pathogen is associated mainly with nosocomial infections and less frequently with community-acquired ones [1]. It is notorious for its antimicrobial resistance against a variety of antibiotic classes, such as beta-lactams, quinolones, aminoglycosides and tetracyclines/glycylcyclines. It possesses a multitude of defence mechanisms: the production of beta-lactamases, especially of serine oxacillinases (Amber class D OXA type) and metallo-beta-lactamases; changes in outer membrane proteins; multidrug efflux pumps; and alterations in the affinity or expression of the Penicillin Binding Proteins. Efflux pumps and the modification of binding sites are also involved in resistance towards quinolones, while efflux- and aminoglycoside-modifying enzymes and 16S rRNA methylation have been employed by the bacterium against aminoglycosides [2]. It does not, therefore, come as a surprise that *A. baumannii* has unfortunately established itself as one of the most significant pathogens of nosocomial infections, with a special predilection for critically ill patients [3]. The Infectious Diseases Society of America has included *A. baumannii* in the ESKAPE group of bacteria. *A. baumannii* is associated with various types of infections ranging from relatively mild, such as urinary tract infections (UTIs), skin and soft tissue infections and tracheitis, to severe, such as Ventilator-associated pneumonia (VAP), bacteraemia (BSI) [associated with Central venous catheter presence or complicating various infections such as VAP and UTIs] and complicated Intra-abdominal infections [4,5]. The main problems are the severe infections as presented in a study by Pogue et al., mainly VAP and bacteraemia due to isolates with resistance to carbapenems [2,3]. 

Various definitions of the resistant profile of Gram-negative bacteria, including *A. baumannii*, have been formulated in order to provide harmony and better understanding of the various clinical trials that address the epidemiology and the clinical effectiveness of antimicrobial agents against these bacteria. The most renowned definitions are those by Magiorakos et al. [6] and by Kadri et al. [7]. Magiorakos et al. defined, for epidemiological reasons, multidrug-resistant (MDR) bacteria as acquired non-susceptibility to at least one agent in three or more antimicrobial categories, extensively drug-resistant (XDR) bacteria as non-susceptibility to at least one agent in all but two or fewer antimicrobial categories (i.e., bacterial isolates remain susceptible to only one or two categories) and Pandrug-resistant (PDR) bacteria as non-susceptibility to all agents in all antimicrobial categories. On the other hand, Kadri et al. proposed a more clinical definition of difficult-to-treat resistance (DTR) as resistance to all beta lactams (including carbapenems) and fluoroquinolones. It is interesting to see that, from Kadri’s original cohort, the bacterium that topped the ranking of DTR was *A. baumannii* with 18.3% DTR in the USA. In other words, for 18.3 percent of *A. baumannii* there was no beta lactam and no quinolone available for treatment. Indeed, mortality was higher for patients with DTR isolates. To provide a better picture of the resistance problem of *A. baumannii* in this study, 29.4% were carbapenem-resistant, 55.4% were resistant to extended-spectrum cephalosporinases and 49.5% were resistant to fluoroquinolones. The situation in Europe and in other parts of the world is no better. While in Northern Europe carbapenem-resistant *A. baumannii* comprises from less than 1% to 10% of the population, in Southern and Eastern Europe this escalates to a staggering ≥50%. A significant *A. baumannii* global burden is evident on other continents with MDR *A. baumannii* prevalence ranging from 64.6% in Eastern Asia to 100% in Central and Latin America with a pool overall mortality rate of 42.6% [8].

One, therefore, understands that to address *A. baumannii* infections is of paramount importance. Guidelines regarding its treatment have been provided from the IDSA [9] and the European Society for Clinical Microbiology and Infection [10]. However, the guidelines do not differentiate data from the last decade from previous data. In our opinion, a focus on the latest data, as in our review, is relevant in regard to current antimicrobial resistance.

## 2. Methods

We sought to identify and analyse data from clinical studies regarding therapeutic options for the treatment of MDR and also XDR Acinetobacter infections during the last decade, 2014–2023. This timeframe also includes the approximately 3 years of the burden of the COVID-19 pandemic, especially the treatment of ICU patients. The Pubmed and Scopus databases were searched. Only clinical trials and articles written in English language regarding the treatment of MDR and XDR *A. baumannii* infections in adult patients (≥18 years) were included. Τhis manuscript does not mean to be an exhaustive review of the literature in general but rather to provide data from the last decade regarding the most relevant studies. Case reports and case series with less than 10 patients were not included.

## 3. Results

We identified a variety of clinical trials from the last decade regarding a variety of antimicrobial agents that are effective against *A. baumannii* infections (Table 1, Table 2, Table 3, Table 4 and Table 5). Among them are first-line agents as described in the definition of DTR, such as beta lactam antibiotics (including beta lactam/beta-lactamase inhibitor combinations and carbapenems), and second-line agents, such as polymyxins, tetracyclines/glycylcyclines, aminoglycosides and trimethoprim/sulfamethoxazole. 

Beta lactams and beta-lactamase inhibitors that have been used in the treatment of MDR, XDR and PDR *A. baumannii* infections are sulbactam, ampicillin/sulbactam and durlobactam/sulbactam, imipenem-cilastatin and meropenem. Antipseudomonal penicillins and cephalosporins such as piperacillin/tazobactam, ceftazidime and cefepime have also been referred to in the literature as possessing in vitro activity but clinical data are scarce. Meropenem and imipenem/cilastatin possess in vitro activity and are also clinically effective, while ertapenem does not. A siderophore cephalosporin, cefiderocol, has FDA approval for treatment of *A. baumannii* infections. 

We present the data existing in the literature during the last decade regarding the use of these specific agents in the treatment of MDR/XDR *A. baumannii* infections and our personal perspective on their use. 

### 3.1. Beta Lactam/Beta-Lactamase Inhibitor Combinations

#### 3.1.1. Sulbactam

Sulbactam, a beta-lactamase inhibitor with in vitro activity against *A. baumannii*, is not usually available as a solitary pharmaceutical agent [11,12]. It is included in various fixed combinations with ampicillin (which does not possess activity against *A. baumannii*) or cefoperazone. Nevertheless, sulbactam is unique as a beta-lactamase inhibitor as it possesses intrinsic antibacterial activity against *A. baumannii* due to its ability to bind Penicillin Binding Proteins 1 and 3. Its clinical effectiveness has been corroborated in various clinical trials. Older studies (non-randomised) have investigated the clinical effectiveness of sulbactam in the treatment of *A. baumannii* infections with a variety of clinical results [13,14,15,16,17,18,19,20]. However, contemporary data by Betrosian et al. [20] have introduced the concept of high-dose ampicillin/sulbactam dosing with the administration of 9 gr every 8 h (i.e., 9 gr of sulbactam daily). Lately, sulbactam has also been combined with another beta-lactamase inhibitor, durlobactam. This pairing of two beta-lactamase inhibitors appears unusual. However, it is based on the fact that durlobactam restores the activity of sulbactam against *A. baumannii*. This is achieved by durlobactam through the inhibition of various class D beta-lactamases (OXA-23, OXA-24 and OXA-58).

A number of clinical trials have been performed mainly investigating the outcome of VAP due to the MDR *A. baumannii* complex being treated with sulbactam, ampicillin/sulbactam, cefoperazone/sulbactam or sulbactam/durlobactam and various comparator antibiotics. We summarise these eleven studies in Table 1. We identified seven non-randomised studies (five retrospective [21,22,23,24,25] and two prospective [26,27]) and four randomised trials [28,29,30,31]. A total of 928 patients were included in these clinical trials. Sulbactam was administered as an ampicillin/sulbactam-containing regimen versus a comparator antibiotic regimen that either did not contain sulbactam or did contain sulbactam with a different comparator antibiotic. The daily dose of sulbactam in these studies ranged from 4 g/day to 8 g/day. As stated above [20], higher doses have been provided in the past that reached 9 g/day and some have also proposed 12 g/day [32]. Nevertheless, the 4–8 g of sulbactam that was administered in the presented trials showed similar results to the comparator medication regarding clinical cure and/or improvement in five trials, better results in four trials and worse results in one trial, while no data were reported in one trial. Regarding mortality, five out of eleven studies showed lower mortality (four with statistical significance and one with a trend) in the sulbactam arm and three studies reported no difference in mortality, while two of the trials did not report data. In total, 58 out of 448 patients that received sulbactam died (mortality of 12.94%), while 162 out of 482 patients in the comparator arm died (mortality 33.6%). Thus, absolute mortality was approximately 20 percent lower in the sulbactam arm than in the comparators. The data from the seven studies that reported on microbiological eradication did not show any statistical difference when comparing sulbactam-containing regimens with comparators.

**Table 1:** Summary of comparative studies examining efficacy of sulbactam in patients with *A. baumannii* infections.

Study (Year)	No. of Evaluable	Infection	Antibacterial Regimens No. Pts				Cure and/or Improvement (%)			Bacteriological Eradication (%)		
			Comparator 1		Comparator 2		Comparator 1	Comparator 2	*p*-Value	Comparator 1	Comparator 2	*p*-Value
21	65	Pneumonia	A/S	22	A/S + V	43	63.6	65.1	0.906	89.5	81.3	0.69
							M:36.4	M:37.2	0.947			
22	98	VAP	A/S	32	COL	66	47	56	0.34	82	52	0.03
							M:9.4	M:25.8	0.07			
							aOR: 6.5 (1.34–31.34) 0.02					
23	84	VAP + B	A/S+I/C	56	TG+I/C	28	NR	NR		NR	NR	
							M:14.3	M:64.3	0.007	NR	NR	
24	106	Pneumonia	C/S	35	CARB	46	71.4	29.3	0.003	NR	NR	
					TG	25		60	0.355			
							M:5.7	M:6.5				
								M:8				
25	130	VAP	C/S	66	TG	42	70	62	0.402	50	33	0.21
					C/S+TG	22	M:5	45	0.208	41	33	0.54
								M:12	0.295 *			
								M:27	0.231 **			
							aOR: 0.115 (0.015–0.89)		0.038			
28	47	VAP	A/S+MR	23	COL+MR	24	69.6	75	0.75	91.3	87.5	0.59
							M:39.13	M:41.67	0.99			
29	23	VAP	A/S+L	12	COL+L	11	83	27	0.007	75	100	NR
							M:41.66	M:81.81	0.04			
26	42	VAP	C/S+TG	21	TG	21	85.7	47.6	0.01	NR	NR	
30	28	VAP	A/S+nCOL	16	COL+nCOL	12	31.2	33.3	NS	43.7	12.5	0.37
							M:16.7	M:37.5	0.22			
27	180	VAP/HAP	A/SorC/S+COL	90	CARB+COL	90	NR	NR	0.658	NR	NR	
							M:51.1	M:55.6				
31	125	Pneumonia	S/D	63	COL	62	62	40	NR	86	61	NR
						M:19		M:32	0.0935 ^¶^			

VAP = Ventilator-associated pneumonia; B = bacteraemia; A/S = ampicillin/sulbactam; C/S = Cefoperazone/sulbactam; CARB = Carbapenem; I/C = imipenem/Cilastatin; COL = Colistin; nCOL = nebulised Colistin; MR= meropenem; TG = tigecycline; L = Levofloxacin; V = Various, i.e., 68.6% Carbapenem, 8.7% cephalosporins, 3.5% Fluoroquinolones; M = mortality; * =comparison between C/S and TG; ** = comparison between TG and C/S+TG; aOR = adjusted Odds Ratio; NR = not reported; ^¶^ = log rank p.

It is evident from the mentioned studies of the last decade that sulbactam not only retained significant clinical effectiveness against *A. baumannii* infections in an era in which antimicrobial resistance became worse on global scale but is also associated with lower mortality against comparator regimens. The new entry of durlobactam/sulbactam holds promise with less potential for nephrotoxicity. The availability of cefoperazone/sulbactam and the data from many Asian countries lend credibility to the role of sulbactam in the treatment of *A. baumannii* infections. 

The present analysis is in full alignment with the IDSA guidelines [9] and the ESCMID guidelines [10] that suggest that when the isolates are susceptible to sulbactam one should consider it as a primary agent for the treatment of *A. baumannii* infections and indeed, interestingly, even if the strains are not susceptible to it. In the studies mentioned above, the sulbactam treatment arm was better or at least equal when compared with various agents, predominantly colistin and tigecycline, in the treatment of multidrug-resistant *A. baumannii* infections.

#### 3.1.2. Cefiderocol

Cefiderocol is a siderophore cephalosporin which has FDA approval for the treatment of *A. baumannii* infections. The unique characteristic of this iron bearing cephalosporin has proved clinically important for Enterobacteriaceae and Pseudomonas aeruginosa as well. Data regarding *A. baumannii* are relatively few. We include the two randomised controlled trials (RCTs) as well as seven non-randomised, mainly retrospective observational, trials reporting on the effect of cefiderocol against a comparator antibiotic in Table 2. It is interesting that in the APEKS-NP study with 312 adult patients with VAP or health-care-associated Gram-negative pneumonia (including 47 due to *A. baumannii*) enrolled, cefiderocol was non-inferior to high-dose, extended-infusion meropenem in terms of all-cause mortality on day 14 [33]. 

Contradicting results were provided by the second RCT, the CREDIBLE-CR [34], regarding the subset of patients with *A. baumannii*, in which 19 out of 39 patients (49%) in the cefiderocol treatment arm died in comparison with 3 out of 17 (18%) in the best available treatment arm. This pointed to a double mortality rate in the cefiderocol treatment arm in patients with nosocomial pneumonia or bloodstream infection due to *A. baumannii*. Obviously, this is a very small sample size to evaluate the effect of any medication, especially as cefiderocol has been licenced by the FDA for *A. baumannii* infections. In addition, one must note that in Acinetobacter-infected patients, 26% had septic shock within a month before or even at randomisation in the cefiderocol group vs. 6% in the best available therapy group. Indeed, 81% of *A. baumanni*-infected patients that were randomised to cefiderocol were in the ICU at randomisation vs. 47% of those that received the comparator. The effect of baseline septic shock on mortality has been presented in a propensity-matched cohort study of 9000 patients with XDR infections [35], which showed a nine-fold higher mortality for patients with septic shock. One has to take this into consideration to evaluate the increased mortality in the cefiderocol arm shown in the CREDIBLE-CR study. 

While the above two studies [33,34] are the only randomised studies until today, there have been a significant number of studies performed in recent years [36,37,38,39,40,41,42], and we present these data in Table 2. Most of these studies were conducted in Italy and are retrospective in nature. A total of 744 patients were included in the studies in the table [33,34,36,37,38,39,40,41,42] that addressed the therapeutic management of VAP and bloodstream carbapenem-resistant *A. baumannii* (CRAB) infections. When examining the mortality of patients that received a cefiderocol-containing regimen versus a comparator regimen that did not contain cefiderocol, mortality was observed in 152 out of the 367 patients (41.4%) that received cefiderocol versus 247 out of the 430 patients (57.4%) that received a comparator antibiotic. These findings suggest that cefiderocol could possibly be associated with lower all-cause mortality than previously thought. The data presented above regarding the potential role of cefiderocol in the treatment of *A. baumannii* infections cannot be ignored. There are conflicting suggestions by the IDSA [9] and ESCMID [10] guidelines regarding the use of cefiderocol in *A. baumannii* infections. Indeed, the IDSA suggests the use of cefiderocol as part of a combination regimen and when non tolerance to other therapeutic agents is present or when no other choices are available. On the other hand, there is a conditional recommendation against the use of cefiderocol in the ESCMID guidelines. We believe that, based on the literature, there is certainly a role for the use of cefiderocol. Obviously, cefiderocol-resistant *A. baumannii* strains are emerging [43]. Nevertheless, this agent retains a significant in vitro activity against *A. baumannii*, as shown by worldwide data [44,45,46,47,48,49]. Indeed, in a survey among 13 infectious disease specialists and 11 anaesthesiologists in Italy regarding the treatment of ICU patients in 87% of cases, cefiderocol was used as an empirical or targeted therapy when *A. baumannii* was the suspected pathogen [50]. 

The most used pharmacological combination when treating MDR *A. baumannii* infections using cefiderocol was fosfomycin (66.7%), followed by colistin (52.4%) and ampicillin/sulbactam (42.9%). The results of the GAME CHANGER trial are awaited to further evaluate the effectiveness of cefiderocol in the treatment of VAP and BSIs due to Gram-negative bacteria (including *A. baumannii*) [51]. 

**Table 2:** Summary of comparative studies examining efficacy of cefiderocol in patients with *A. baumannii* infections.

Study (Year)	No. of Evaluable	Infection ^¶^	Antibacterial Regimens No. Pts				Cure and/or Improvement (%) Mortality (%)			Bacteriological Eradication (%)		
			Comparator 1		Comparator 2		Comparator 1	Comparator 2	*p*-Value	Comparator 1	Comparator 2	*p*-Value
33	47	Pneumonia	CFD	23	MR	24	52	58	NS	39	33	NS
							M:19	M:22	NS			
34	54	Pneumonia/B/UTI	CFD	37	BAT	17	43	53	NS	27	29	NS
							M:49	M:18	NR			
36	107	B:27 LRTI:14 ALL COVID-19	CFD	42	COL−R	65	40	36	0.45	28	21	0.24
							M:55	M:58	0.70			
37	124	B:79 VAP:35	CFD−R	47	COL−R	28	NR	NR				
							M:34%	M:55.8	0.018 *	82.6	93.2	0.079
38	111	B:53 P:47	CFD−R	60	COL−R	51	73	67	0.44	43	41	0.82
							M:51	M:37				
39	118	B	CFD−R	43	COL−R	75	NR	NR		NR	NR	
							M:40	M:59	0.045			
40	121	VAP ALL COVID-19	CFD−R	55	NON CFD−R	66	NR	NR		47	69	0.038
							M:44	M:67	0.011			
41	90	VAP	CFD+Ncol	40	COL+nCOL	50	75	52	0.02	70	40	0.003
							M:35	M:52	NS			
42	73	Bacteremic VAP ALL COVID-19	CFD−R	19	COL−R	54	NR	NR		NR	NR	
							M:31.5	M:98.1	<0.001			

Abbreviations: No. = number; Pts = patients; M = mortality; ^¶^ = numbers in the infection column are absolute numbers of patients with the specific type of infection mentioned; VAP = Ventilator-associated pneumonia; *p* = pneumonia; LRTI = lower respiratory tract infection; B = bloodstream infection; CFD = cefiderocol; CFD-R = cefiderocol-containing regimen; COL-R = colistin-containing regimens, i.e., combination regimens ± monotherapy; COL = Colistin; nCOL = nebulised colistin; * = regarding mortality of bacteraemia only; MR= meropenem; BAT = best available treatment; NR = not reported; NS = non-significant.

#### 3.1.3. Polymyxins

Polymyxins have been at the forefront of treatment for carbapenem-resistant *A. baumannii* infections for approximately the last 20 years [52]. The two polymyxins used are colistin (polymyxin E) and polymyxin B. The necessity of using them originated form the lack of suitable alternative antibiotics [53,54,55,56,57,58,59,60,61]. Polymyxins were used, though, before their re-emergence in the treatment of infections due to non-fermenters in patients with cystic fibrosis. While nephrotoxicity is a relative concern of their use, this is usually reversible and should not deter physicians from prescribing these drugs. Although most of the studies reporting on the clinical use of colistin in the past were non-randomised, their effectiveness and safety are undisputable. We summarise non-randomised studies performed in the last decade regarding the use of colistin [62,63,64,65,66,67,68,69,70,71] in Table 3 and randomised comparative studies [72,73] in Table 4. It is obvious from the data presented that colistin retains significant clinical effectiveness and favourable outcomes on decreased mortality even as a monotherapy. Combinations with sulbactam or tigecycline seem to confer a benefit. Indeed, in the non-randomised studies mortality was similar in patients receiving colistin as a monotherapy (47.1%, 302 out of 642 patients) in comparison to combination therapy (44.9%, 376 out of 836 patients). Mortality was also similar in the randomised studies when comparing patients receiving colistin monotherapy (44.6%, 167 out of 374 patients) with patients receiving combination therapy (46.3%, 172 patients out of 371). 

**Table 3:** Summary of non-randomised comparative studies examining efficacy of polymyxins in patients with *A. baumannii* infections.

Study (Year)	No. of Evaluable	Infection	Antibacterial Regimens No. Pts				Cure and/or Improvement (%)			Bacteriological Eradication (%)		
			Comparator 1		Comparator 2		Comparator 1	Comparator 2	*p*-Value	Comparator 1	Comparator 2	*p*-Value
62	250	B	COL	36	COL+V	214	30.6	46.3	0.19	55.6	79.9	0.001
							M:44.5	M:31.8	0.14			
63	89	VAP	COL	57	COL+S	32	29.8	40	0.50	72.3	85.7	0.28
							M:51.9	M:73	0.53			
64	79	VAP SSTI, UTI	COL	46	COL+V	23	NR	NR		NR	NR	
							M:26.1	M:26.08	NS			
65	55	B	COL+C	26	COL+TG	29	NR	NR		100	82	
							M:15	M:35	0.1	NR	NR	
66	70	VAP	COL	13	COL+S	20	76.5	55	0.53	52.9	63.6	0.16
					COL+C	33		63.6	0.35		60	0.23
							M:41.2	M:70	0.21			
								M:48.5	0.5			
67	107	B	COL	36	NON−COL	71	77.1	77.1	0.45	69	83	0.13
							M:47.2	M:52.77	0.36			
68	118	B	COL	76	COL+TG	42	NR	NR		NR	NR	
							M:22	M:24	1			
69	39	VAP	COL	19	COL+A/S	20	15.8	70	0.001	33.3	71.4	0.19
							M:63	M:50	NS			
70	71	B	COL	40	COL+MR	31	40	61.3	0.07	NR	NR	
							M:47.5	M:25.8	0.08			
71	617	NP	COL	293	NON−COL	324	aOR: 1.27 (0.92–1.75) ^¶^			aOR: 3.44 (2.36–5.02) ^¶^		
							M:54.3 *	M:53.7 *	0.87			

B = bloodstream infection; VAP = Ventilator-associated pneumonia; SSTI = skin and soft tissue infection; NP = nosocomial pneumonia; COL = Colistin; V = Various; S= sulbactam; TG = tigecycline; C = Carbapenem; NON-COL= regimen not containing colistin; A/S = ampicillin/sulbactam; MR= meropenem; M = mortality; aOR = adjusted Odds Ratio; NR = not reported; ^¶^ = in favour of colistin. * Patients with multiple organ failure and SOFA score more than 7 treated with colistin-based regimen had better survival (aOR: 0.38) than non-colistin regimens (aOR: 0.52), *p* = 0.011.

**Table 4:** Summary of randomised comparative studies examining efficacy of polymyxins in patients with *A. baumannii* infections.

Study (Year)	No. of Evaluable	Infection	Antibacterial Regimens No Pts				Cure and/or Improvement (%)			Bacteriological Eradication (%)		
			Comparator 1		Comparator 2		Comparator 1	Comparator 2	*p*-Value	Comparator 1	Comparator 2	*p*-Value
73	312	VAP B, UTI	COL	151	COL+M	161	21	27	0.643	69	65	0.489
					M:46	M:52	0.404					
72	413	VAP, B W, UTI	COL	213	COL+M	210	32	40	NS	63	57	NS
					M:46	M:42	NS					

VAP = Ventilator-associated pneumonia, B = bloodstream infection, W = wound infection, UTI = urinary tract infection, COL = Colistin, M = meropenem, M = mortality, NS = non-significant.

Non parenteral administration of colistin has also been reported. Nebulised colistin has been used mainly as an adjunctive to intravenous treatment and rarely as a monotherapy without providing any significant benefit in the majority of studies, although data are scarce and mostly older than the timeframe we elected to evaluate [74,75]. While a potential drawback is bronchospasm in some patients, the medication has also been used in children in its aerosolised form, without any major safety concern in patients with and without cystic fibrosis. Intrathecal or intraventricular colistin has a significant role in the treatment of neurosurgical infections [76].

Among polymyxins, polymyxin E (colistin) is used more frequently. Nevertheless, there are data in the literature regarding the use of polymyxin B. Polymyxin B is mostly used in North and South America and Asia. While, generally, polymyxin B has been shown in various studies to be less nephrotoxic than colistin, a recent meta-analysis showed a similar nephrotoxicity. We identified a significant number of studies during the last decade pointing to the clinical and microbiological effectiveness and safety of polymyxin B as a treatment for *A. baumannii* infections [77,78,79,80,81,82,83,84,85,86,87,88,89,90,91,92,93,94,95,96,97,98,99]. Indeed, the agent is equally effective with colistin and relatively safer in regard to nephrotoxicity [100,101,102]. 

#### 3.1.4. Tetracyclines and Glycylcyclines

Tigecycline retains significant in vitro activity against *A. baumannii* [103] and has been used in the treatment of infections caused by this organism. However, tigecycline, as with all antibiotics, is not immune to antimicrobial resistance mechanisms [104]. In addition, pharmacokinetics regarding blood levels have shown that the levels achieved are not adequate for the treatment of *A. baumannii* bacteraemia [105]. Furthermore, the same holds true for the levels in the lungs [106,107]. It is therefore not surprising that higher doses (indeed, double doses) are suggested for use in clinical practice [108,109]. Therefore, high-dose tigecycline has been suggested (200 mg loading dose, followed by 100 mg bid) and indeed has been found more effective than standard-dose tigecycline [108,109]. We identified seven retrospective studies [110,111,112,113,114,115,116] during the last decade (Table 5) that compared tigecycline vs. a comparator antibiotic, colistin in five and sulbactam in two. In all, tigecycline was administered in the standard dosing, i.e., a 100 mg iv initially and then 50 mg twice daily. In total, 182 out of the 374 patients that received tigecycline died (48.6%) versus 200 out of the 508 (39.3%) in the comparator arm. Breaking it down, mortality was better in the tigecycline arm in one study, worse in two and without statistical difference in three. One must note that in one of these studies, which only enrolled patients with bloodstream infections [116], mortality was 22% higher in the tigecycline arm.

**Table 5:** Summary of comparative studies examining efficacy of tigecycline in patients with *A. baumannii* infections.

Study (Year)	No. of Evaluable	Infection	Antibacterial Regimens No. Pts				Cure and/or Improvement (%)			Bacteriological Eradication (%)		
			Comparator 1		Comparator 2		Comparator 1	Comparator 2	*p*-Value	Comparator 1	Comparator 2	*p*-Value
110	88	VAP	TG	44	COL	44	NR	NR		NR	NR	
							M:60.7	M:44	0.04			
111	55	VAP, B W, UTI	TG	16	COL	39	43.8	48.7	0.737	12.5	46.2	0.049
							M:56.3	M:43.6	0.393			
112	79	VAP	TG+COL	19	COL+I	60	NR	NR		NR	NR	19
						M:26.3	M:53.3	0.04				
113	70	VAP±B	TG−R	30	COL−R	40	47	48	0.95	23	30	0.54
						M:33	M:30	0.77				
114	168	VAP	TG−R	84	SUL−R	84	66.7	66.7	1	33.3	63.5	<0.0001
						M:25	M:17.9	0.25				
115	212	VAP	nCOL+TG	106	nCOL	106	NR *	NR *		NR	NR	
						M:34	M:22.6	0.17				
116	210	B	TG	75	C/S	135	NR	NR		NR	NR	
							M:51.9	M:29.3	0.001			

VAP = Ventilator-associated pneumonia, B = bloodstream infection, W = wound infection, UTI = urinary tract infection, I = imipenem/Cilastatin, TG = tigecycline, COL = Colistin, nCOL = nebulised Colistin, M = mortality, NR = not reported, NR * = lack of TGC therapy (aHR = 0.52; 95% CI = 0.27–1.00; *p* = 0.049) adversely influenced the 14-day clinical cure.

#### 3.1.5. Minocycline

There are relatively few data points regarding the use of minocycline against *A. baumannii* infections. Minocycline is an antibiotic belonging to the tetracyclines group and has been used in the therapeutic arena for over 6 decades. Its use is indicated in acne and in a variety of infections cause by aerobic Gram-positive and Gram-negative microorganisms, intracellular microorganisms and, interestingly, also against *A. baumannii* [117,118]. In a meta-analysis, minocycline was reported to have been used in the treatment of *A. baumannii* infection in 228 patients [119]. The clinical and microbiological success rates reported following minocycline treatment were 72.6% and 60.2%, respectively. Nevertheless, in the vast majority (~92% of these patients) it was part of a combination regimen, mainly with carbapenems or colistin. Only a handful of studies have been conducted in the last decade [120,121,122,123,124] that support the use of minocycline in the treatment of A. baumanni infections. Susceptibility to minocycline seems to be correlated with the absence of the TeTB gene [125]. 

#### 3.1.6. Trimethoprim/Sulfamethoxazole (TMP/SMX)

There is only one retrospective cohort study, by Raz et al., that included 53 patients receiving TMP/SMX for severe infections caused by CRAB who were matched with 83 patients treated with colistin or ampicillin/sulbactam [126]. A variety of infections were present in these patients: pneumonia (71.3%) was the most frequent, followed by skin and soft tissue infections, UTIs and central line-associated bacteraemia. All-cause 30-day mortality was lower with TMP/SMX compared with the comparator antibiotics among all patients (24.5% vs. 38.6%, *p* = 0.09) and in the propensity score-matched subgroup (29% vs. 55.2%, *p* = 0.04). 

#### 3.1.7. Fosfomycin and Rifampicin

Fosfomycin and rifampicin are antibiotics that have no satisfactory clinical effectiveness against *A. baumannii*. However, there are scarce data in the literature supporting their role as part of combination regimens with other antimicrobial agents. 

Sirijatuphat and Thamlikitkul randomised 94 patients infected with carbapenem-resistant *A. baumannii* to receive colistin alone or colistin plus fosfomycin for 7 to 14 days [127]. A survival analysis showed no significant difference between the patients who received combination therapy and monotherapy. However, microbiological eradication rates in the combination group were significantly higher than those in the monotherapy group, 100% versus 81.2%, at the end of study treatment. 

Russo et al. performed a prospective study including 44 patients with hospital-acquired pneumonia due to MDR-*A. baumannii* strains [128] that were treated with fofomycin combinations. The following combinations were administered: fosfomycin plus colistin in 11 (25%) patients, fosfomycin plus carbapenem plus tigecycline in 8 (18.2%), fosfomycin plus colistin plus tigecycline in 7 (15.9%), fosfomycin plus rifampin in 7 (15.9%), fosfomycin plus tigecycline in 6 (13.6%), fosfomycin plus carbapenem in 3 (6.8%), and fosfomycin plus aminoglycoside in 2 (4.5%). Cox regression analysis showed that a fosfomycin-containing regimen was associated with 30-day survival (HR 0.04, CI 95% 0.01–0.13, *p* < 0.001).

Regarding rifampicin, we identified only one RCT in the time period we selected to evaluate [129] with a very small sample size of nine patients with pneumonia caused by colistin-resistant *A. baumannii*. In this study, patients were randomised to colistin/rifampicin combination therapy or colistin monotherapy. Unsurprisingly, this study failed to show any difference in clinical and microbiological outcomes. Prior reports, such as those by Durante-Magnoni et al. [61] and Aydemir et al. [130], also questioned the clinical significance of adding rifampicin to colistin. 

### 3.2. Newer Beta-Lactamase Inhibitors

There are no clinical trials regarding the clinical effectiveness of the newer beta-lactamase inhibitors (avibactam, relebactam, varbobactam).

## 4. Discussion

From the studies evaluated, it is suggested that for MDR/XDR *A. baumannii* isolates, which usually are resistant to beta-lactams (including carbapenems) and quinolones, one has to resort to high-dose sulbactam (6–9 g daily), colistin (intravenous ± nebulised), or polymyxin B or high-dose tigecycline (i.e., 100 mg bid). 

High-dose sulbactam was initially suggested in 2008. The data from the last decade presented herein (Table 1) corroborate its effectiveness in clinical practice. It is evident from the mentioned studies from the last decade that sulbactam not only retained significant clinical effectiveness against *A. baumannii* infections in an era in which antimicrobial resistance became worse on a global scale but is also associated with lower mortality against comparator regimens. Indeed, mortality was approximately 20 percent less in patients that received sulbactam than the comparator. The new entry of durlobactam/sulbactam holds promise with less potential for nephrotoxicity. The availability of cefoperazone/sulbactam and the data from many Asian countries lend credibility to the role of sulbactam in the treatment of A. baumannii infections. 

The present analysis is in full alignment with the IDSA guidelines [9] and the ESCMID guidelines [10] that suggest that when the isolates are susceptible to sulbactam one should consider it as a primary agent for the treatment of *A. baumannii* infections and indeed, interestingly, even if the strains are not susceptible to it. In the studies mentioned above, the sulbactam treatment arm was better or at least equal when compared with various agents, predominantly colistin and tigecycline, in the treatment of multidrug-resistant *A. baumannii* infections.

Therefore, the ESCMID and IDSA guidelines that view sulbactam as a first-line option for MDR/XDR *A. baumannii* isolates are, in our opinion, more than justified. As sulbactam is usually found in parenteral form in various combinations with other antimicrobials, such as ampicillin (ampicillin 1 g combined with 0.5 g of sulbactam, ampicillin 2 g combined with 1 g of sulbactam), cefoperazone (cefoperazone 1 g combined with 0.5 g of sulbactam, cefoperazone 1 g combined with 1 g of sulbactam or cefoperazone 2 g combined with 1 g of sulbactam) or durlobactam, one has to calculate the amount of sulbactam necessary to administer to the patient.

Based on the data from the literature presented, polymyxin E (colistin) and polymyxin B retain their crucial role in the treatment of severe *A. baumannii* infections. Indeed, in the majority of studies, polymyxins fared equally well as monotherapies when compared with combination regimens (Table 3 and Table 4). While the vast majority of studies in the literature do not support a combination regimen of these agents, both the IDSA and the European guidelines advise in favour of such a combination. Indeed, the IDSA and European guidelines state that it is preferable to use combinations of antimicrobial agents to which susceptibility testing has been performed and found relevant. We concur, in view of the severity of these infections (patients are usually critically ill), the possibility of the emergence of resistance, the high bacterial quantities that predate targeted antibiotic therapy (i.e., prior to bacterial culture and susceptibility results) and because of suboptimal doses of colistin (at times, the prescribed dose was half of what should be given), that the use of combination regimens is justified and safe. However, further data from high-quality RCTs are much needed in order to corroborate or negate the theoretical advantage of a combination regimen. While polymyxin B is favoured in general in the guidelines, based on our experience [57] and the data from the literature [62,63,64,65,66,67,68,69,70,71,72,73] we do not believe that colistin is inferior to polymyxin B in terms of clinical effectiveness. We acknowledge the smaller degree of nephrotoxicity associated with polymyxin B. Nephrotoxicity is a potential adverse event of colistin treatment but it is usually reversible and should not prevent clinicians from prescribing the drug [131]. Adjustments of colistin dosing have been agreed upon in consensus meetings of scientific associations and provide useful guidance for adjustments based on creatinine clearance measurements [132]. We therefore believe that colistin should not be stricken so easily from the therapeutic armamentarium and just preferred for use in UTIs over polymyxin B, especially as minocycline and polymyxin B are not available in parenteral forms worldwide. 

Regarding tigecycline, one must be aware that the standard dose of tigecycline (i.e., a 100 mg loading dose iv followed by a 50 mg bid) does not achieve adequate levels in the blood and possibly in the respiratory system to combat MDR/XDR *A. baumannii* [104,105,106,107], and this translates to worse clinical outcomes in clinical practice in comparison to comparators. Therefore, based on the findings of the comparative studies presented in Table 4, we suggest that standard dosing of tigecycline should be avoided for the treatment of *A. baumannii* infections and only high-dose tigecycline should be employed. On the other hand, high-dose tigecycline (a 200 mg loading dose followed by a 100 mg bid) provides a useful antimicrobial agent in combination with one of the other agents active against carbapenem-resistant *A. baumannii* (i.e., colistin or polymyxin B or high-dose sulbactam) [108,109]. 

A crucial issue is also the use of cefiderocol. The European guidelines advise against the use of cefiderocol as a treatment for *A. baumannii* infections, while the IDSA guidelines advise its use on a conditional basis provided that there are no alternatives or there is intolerance to other antimicrobials. The IDSA guidelines advise that cefiderocol, if used, is to be part of a combination regimen with caution until and if more favourable data are available. Their recommendation is mainly based on the data provided by the RCT from Basseti et al., which had a very small number (54) of patients with *A. baumannii* infections that disfavoured cefiderocol treatment, and those by the APEKS-NP, which showed no difference. We could not agree more with the IDSA guidelines on this issue that caution is needed. Nevertheless, there is already a significant number of trials (although non-randomised) presented herein that are in favour of cefiderocol. Indeed, the comparative studies by Pascale et al. [36], Falcone et al. [37]., Mazzitelli et al. [38], Bavaro et al. [39], Rando et al. [40], Dalfino et al. [41] and Russo et al. [42] included 637 patients, and 306 of them received cefiderocol for *A. baumannii* infections with favourable clinical outcomes. 

Aminoglycosides are proposed as first-line agents by both the IDSA and ESCMID in the treatment of carbapenem-resistant *A. baumannii*. Nevertheless, there are no data in the current literature to support aminoglycoside monotherapy in severe infections such as VAP and bacteraemia. Therefore, they must not be given as a monotherapy for these severe *A. baumannii* infections. Aminoglycosides can be used in these types of infections as a part of combination regimens. 

Regarding fosfomycin and rifampicin, no convincing clinical evidence exists regarding their use, and it seems that their role in the treatment of *A. baumannii* infections remains to be clarified by larger trials. 

## 5. Conclusions

In conclusion, one hopes for additional trials, especially randomised trials, that will further clarify the ever-lasting challenge of *A. baumannii* infections’ treatment. While prevention through infection control measures is of paramount significance, a clear understanding of the current literature is mandatory to provide suitable therapeutic management to critically ill patients with *A. baumannii* infections. Hence, based on our review, one has to resort to at least one of the following, sulbactam, polymyxins E or B, tigecycline or aminoglycosides, against multidrug-resistant (MDR) or extensively drug-resistant (XDR) *A. baumannii* infections. Furthermore, cefiderocol is a useful addition in the fight against *A. baumannii* infections.

## Data Availability

Data are available through the Pubmed and Scopus databases.
